# Factors associated with infants’ and young children’s (6–23 months) dietary diversity in Pakistan: evidence from the demographic and health survey 2012–13

**DOI:** 10.1186/s12937-017-0297-7

**Published:** 2017-12-06

**Authors:** Sarosh Iqbal, Rubeena Zakar, Muhammad Zakria Zakar, Florian Fischer

**Affiliations:** 1 Institute of Social and Cultural Studies, University of the Punjab, P.O. Box 54590, Lahore, Pakistan; 2 Department of Public Health Medicine, School of Public Health, Bielefeld University, P.O. Box 100 131, 33501 Bielefeld, Germany

**Keywords:** Dietary diversity, Children, Infant, Pakistan

## Abstract

**Background:**

Optimum nutrition and good feeding practices amongst infants and young children are the key determinants of growth for a healthy life. Dietary diversity is considered to be a reliable and easy-to-measure proxy variable to assess young children’s feeding practices for dietary adequacy and nutritional intake. This research aims to examine the current practices of dietary diversity amongst infants and young children aged 6–23 months in Pakistan and the various associated factors at child, maternal, household and community levels.

**Methods:**

Secondary data analysis was performed for this research using the nationally representative dataset of the Pakistan Demographic and Health Survey 2012–13. Data on the last-born infants and young children aged 6–23 months (*n* = 1102) was taken from their mothers’ interviews, who reported on their child’s consumption of 7 food groups during the 24 h immediately preceding the survey. Data was analysed, using IBM® SPSS® Complex Sample to measure the association between children’s dietary diversity and various factors at child, maternal, household and community levels through multiple linear regressions.

**Results:**

Our research uncovered positive associations between children’s dietary diversity and other sociodemographic variables. Overall, a variation was observed in consumption of 7 food groups across the youngest, middle and oldest age-groups of children. Multivariate analysis revealed that the children’s Dietary Diversity Score (scale from 0 to 7) increases to 0.56 (95% CI: 0.18–0.94) amongst children in the middle age-group (12–17 months). Furthermore, the children who were still breastfeeding, with mothers who had a primary level of schooling and whose mothers also received information/services from lady health workers (LHWs) on maternal and child health were found to be a statistically significant predictor of infants’ and young children’s dietary diversity. Nevertheless, amongst them, the DDS had a negative association with the children’s status of still breastfeeding and mothers’ primary level of schooling, whereas it had a positive association with children being in the middle age-group and with mothers who received information/services from LHWs.

**Conclusion:**

The dietary diversity of infants and young children aged 6–23 months has a modest, nevertheless statistically significant, relationship with sociodemographic characteristics in Pakistan. There is a need for practical efforts to change the behaviour of communities to encourage more diverse foods to promote the healthy growth of children.

## Background

Optimum nutrition and good feeding practices amongst infants and young children are the key determinants of growth for a healthy life [1, 2]. The period from birth to two years of age is considered critical to avoid micronutrient deficiencies and common childhood illnesses. Therefore, a good-quality diet with adequate nutrient intake is fundamental, along with breastfeeding, starting from the first 6 months after birth, to meet the nutritional requirements of young children [3, 4]. Hence, inappropriate feeding practices may cause malnutrition, leading to stunting, wasting and being underweight amongst infants and young children. The incidence of malnutrition is highest amongst young children aged 6–18 months in most developing countries, and it is difficult to compensate for this later in childhood [5].

Malnutrition is a predominant public health challenge in Pakistan [6]. It has the world’s sixth largest population [7] and is a lower-middle-income country [8], where approximately a quarter of the population is unable to meet their nutritional requirements (i.e., 2350 cal per adult per day) [9]. Malnutrition is deeply rooted in poverty and food insecurity, due to which individuals lack access to adequate quality food, due to non-availability, inequitable distribution, insufficient purchasing power, and/or inadequate use of diverse foods at a household level [10]. According to the National Nutrition Survey (NNS) of Pakistan [11], more than half of the country’s population is food insecure. Additionally, the NNS also revealed that the low rate of breastfeeding and inadequate complementary feeding practices are the major causes of malnutrition among infants and young children [11].

Dietary diversity has long been recognised as an essential component of an adequate and high-quality diet for young children [12]. A diverse diet entails the consumption of a variety of food items and/or food groups, increasing the probability of nutrient adequacy among children and promoting their physical and cognitive development [13, 14]. Dietary diversity is considered a reliable and easy-to-measure proxy variable to assess young children’s feeding practices for dietary adequacy and nutritional intake, as suggested by the World Health Organisation (WHO) [15–19].

Lack of dietary diversity is common in poor populations, as their diets are predominately based on starchy staples with few or no meat/animal products and only seasonal vegetables and fruits [16]. It is evident that dietary diversity is strongly associated with demographic and socioeconomic characteristics [16]. Rationally, this seems plausible as families from urban areas have greater incomes and are thus inclined to eat more diverse foods, because of their better access to resources and exposure to beneficial environmental conditions [20]. Moreover, there are varied and complex cultural influences which are unique to different societies, affecting children’s feeding practices [21, 22]. For example, in the case of Pakistan, various nutritious foods are not given to infants and young children due to the misconception that they will cause illness [23] or that children will not be able to digest food other than breastmilk [24].

Previous research has explored the association between dietary diversity and household-level socio-economic characteristics [16, 20, 21, 25]. However, there is a paucity of such research in Pakistan, where 12.4% of the population lives below the poverty line [26] and the overall dietary quality is likely to be poor [27]. Thus, this article aims to examine the current dietary diversity amongst Pakistani infants and young children aged 6–23 months and the various associated factors at child, maternal, household and community levels.

## Methods

### Study design

This research is cross-sectional in nature and used the nationally representative dataset of the Pakistan Demographic and Health Survey (PDHS) carried out in 2012–13 [28]. The choice of PDHS 2012–13 is based on the fact that it is the most recent publicly available dataset and contains information about all the necessary dietary diversity and child-feeding variables required for this research. The PDHS 2012–13 was conducted as part of the Measure Demographic and Health Surveys Project undertaken by ORC Macro with the financial support of the US Agency for International Development. It used a multistage cluster sampling design for data collection on multiple maternal and child-health-related indicators. The sampling design was developed by the National Institute of Population Studies (NIPS) and the Pakistan Bureau of Statistics. In the first stage, sampling areas of 248 urban and 252 rural units were selected, while in the second stage, households were selected through a systematic random sampling technique. A total of 14,000 households, comprising 6944 from urban areas and 7056 from rural areas, were randomly selected. NIPS staff completed the fieldwork between October 2012 and March 2013, almost a month after the rainy season [28]. A total of 20 field teams collected data, each consisting of a supervisor, a field editor, one male and three female interviewers. Data quality was ensured through quality-control interviewers, field coordinators and senior NIPS team members. Along with the fieldwork, the data processing was initiated simultaneously [28]. Completed questionnaires were edited, entered immediately in the field using the computer-assisted field editing system and also uploaded on the same day by the field editor. Secondary editing of the completed questionnaires was carried out in the NIPS office, where all the data was entered twice for 100% verification using the CSPro computer package [28].

The PDHS 2012–13 used four types of questionnaires for data collection: Household Questionnaire, Woman’s Questionnaire, Man’s Questionnaire, and Community Questionnaire. The standard women’s questionnaire was administered to ever-married women of reproductive age (15–49 years). Of the eligible women, a total of 13,558 ever-married women were successfully surveyed through face-to-face interviews, giving a response rate of 93% [28]. The component of child health was part of the woman’s questionnaire, which was administered only to women who had given birth to at least one child during the last five years preceding the survey, consisting of 7461 women [28]. Out of these, the research selected mothers having a last-born child aged 6–23 months. Hence, this analysis was limited to the youngest infant and child aged 6–23 months, yielding a sample size of 1102 children. PDHS 2012–13 used a 24-h recall method to collect data on infant and young children’s feeding practices through interviews with mothers having a last-born child below 2 years of age [28]. During the interview, the mothers recalled multiple food items consumed by their child over the previous 24 h. NIPS field teams (interviewers) noted the responses against the food items available in the PDHS dataset, which were later grouped into 7 food groups, as recommended by the WHO [19]. Any food items mentioned that were not on the list were recorded as ‘other foods’. Once the mother had finished recalling the food items given to the child, the interviewers read out those food items from the questionnaire that had not been mentioned earlier by mothers in order to facilitate their recall [4, 19, 28].

### Variables

#### Outcome variable

The Dietary Diversity Score (DDS) is the outcome variable for this research. The DDS is calculated using a 24-h recall method of food items/groups available in the PDHS dataset. It is the sum of the various food items consumed by a child (6–23 months old) over the 24 h immediately preceding the interview with mothers, who reported on the child’s food consumption, as mentioned earlier. Dietary diversity ranges from 0 to 7, and categorises all food items into 7 food groups, as recommended by the WHO [4, 19]. These are: (1) staples (grains/cereals, roots and tubers), (2) legumes and nuts, (3) dairy products (milk, yogurt and cheese), (4) flesh foods (meat, fish, poultry and liver/organ meats), (5) eggs, (6) vitamin A-rich fruits and vegetables; and (7) other fruits and vegetables. A score of 1 was assigned to each of the above-mentioned food groups given to a child. Thus, the DDS gives an accumulated score with a maximum of 7 and is analysed as a continuous variable. In cases where any of the food items consumed by the child did not fit into the above food groups as recommended by the WHO, these were labelled ‘other foods’.

#### Sociodemographic variables

The various sociodemographic factors were measured at four levels: child, maternal, household and community.

*Child-level* factors included both continuous and categorical variables, such as birth order as continuous variables and child’s age in months (6–11 months, 12–17 months and 18–23 months), child’s sex (male/female), size of child at birth (small or <average, average or >average, very large) and whether the child is still breastfed or not, as categorical variables.

The *maternal-level* factors included three continuous variables: age at first birth, parity (number of children ever born) and empowerment/autonomy. The mother’s empowerment index was constructed based upon her participation in various decisions, including final say in making household purchases, final say on how to spend money earned by herself and her spouse, final say on her own healthcare and final say on visiting family or relatives. The possible responses where the woman has “a say at all” (either alone or jointly with the husband/partner or jointly with another person) were combined together. Moreover, the mothers’ opinions or attitudes towards domestic violence [29] on the number of circumstances that justified beating a wife (here referred to as a mother) were collected: a) going out without telling spouse, b) neglecting the children, c) arguing with spouse, d) refusing to have sex with spouse, and e) burning the food. Thus, the empowerment index was generated by combining mothers’ participation in decision-making and their attitudes towards domestic violence. Details of the construction of the empowerment index are published elsewhere [30].

The other maternal factors were categorical variables, specifically: mother’s age in years (15–24 years, 25–34 years, 35 years and above), level of education (no education, primary, secondary, or higher level), employment status (employed vs. unemployed), number of antenatal care visits conducted (at least 4 visits vs. less than 4 visits) and information/services received from a Lady Health Worker (LHW) on maternal and child health (yes vs. no).

The *household-level* factors included the number of children under 5 years old and the categorical variables of exposure to mass media, including TV, radio, newspapers (yes vs. no), and wealth quintiles (measured on the basis of household assets and ownership of a number of consumer items and divided into five quintiles: richest, richer, middle, poorer, poorest) [31], while the *community-level* characteristics included the place of residence (urban vs. rural) and region (Punjab, Sindh, Balochistan, Khyber Pakhtunkhwa, Islamabad or Gilgit Baltistan).

### Statistical analysis

We performed the data analysis using IBM® SPSS® Complex Sample version 21. Sampling weights were calculated. Descriptive statistics were used to estimate the arithmetic means and standard error (SE) for continuous variables, while frequencies (f) and percentages (%) were examined for categorical variables. A simple linear regression analysis was applied to examine the association between dietary diversity and multiple factors involved at the child, maternal, household and community levels. Afterwards, a multiple linear regression was conducted using those variables that were found to be significantly associated with infants’ and young children’s dietary diversity in the bivariate analysis. In the multiple linear regression, three models were constructed. Model A examines the relationship between dietary diversity and child-level characteristics, including age and whether the child is still breastfed or not. In Model B, the maternal level was also added, such as the mother’s age, age at first birth, level of education, number of ANC visits received and information/services received from LHWs on maternal and child health. For Model C, in addition to child and maternal-level factors, household and community-level characteristics were also included, such as number of children under 5 years old and place of residence.

An association was considered statistically significant in cases where *p* ≤ 0.05. Moreover, the multicollinearity was also calculated using a variance inflation factor (VIF) in the coefficiency estimates of the linear regression. VIF values greater than 2.5 were considered indicative of multicollinearity [32]. No multicollinearity was observed in the regression analysis.

## Results

### Characteristics of dietary diversity

Table 1 shows the food items, categorised into 7 food groups as per WHO recommendations, consumed by 6–23-month-old children during the 24 h immediately preceding the survey. The findings revealed that most of the children (75.2%) consumed staples (grains/cereals, roots and tubers), while around 50% were given dairy products (milk, yogurt and cheese). Around 33% of children had other fruits and vegetables, about quarter had eggs, and about 20% children had flesh (meat, fish, poultry and liver/organ). However, very few children (7.94) consumed legumes (beans, peas, lentils) or nuts. A variation was observed regarding consumption of the 7 food groups across different age-groups of children, between 6 and 11 months, 12–17 months and 18–23 months. Findings revealed that the middle age-group, aged 12–17 months, consumed more food groups than the oldest (18–23 months) or youngest (6–11 months) age-groups. For example, 82% of children from the middle age-group, 77% from the oldest and 67% from the youngest age-group had eaten staples (grains/cereals, roots and tubers) during the 24 h preceding the survey. The same pattern can be seen in other food groups as well.

**Table 1:** Frequency and percentage of 6–23 months old infant and young children consumed different food groups by age-group of children (*n* = 1102) from Pakistan Demographic and Health Survey 2012–13

Food groups consumed^a^	Infant and young children aged 6–23 months (*n* = 1102)		Age-group of children					
			Youngest children aged 6–11 months (*n* = 400)		Middle-age children 12–17 months (*n* = 407)		Oldest children aged 18–23 months (*n* = 295)	
	f^b^	%^b^	f^b^	%^b^	f^b^	%^b^	f^b^	%^b^
Staples (grains/cereals, roots and tubers)	829	75.2	269	67.3	333	81.8	227	76.9
Legumes (beans, peas, lentils) and nuts	82	7.4	7	1.8	48	11.8	27	9.2
Dairy products (milk, yogurt and cheese)	557	50.5	182	45.5	222	54.5	153	51.9
Flesh foods (meat, fish, poultry and liver/organ meats)	211	19.1	48	12.0	78	19.2	85	28.8
Eggs	251	22.8	65	16.3	111	27.3	75	25.4
Vitamin A-rich fruits and vegetables	206	18.7	42	10.5	91	22.4	73	24.7
Other fruits and vegetables	373	33.8	105	26.3	161	39.6	107	36.3

^a^Food groups consumed by the children during 24 h prior to the survey

^b^f denotes to the frequency and % shows percentages of the values given

### Characteristics of the sample

Tables 2 and 3 present the descriptive statistics for the variables used in this research. In Table 2, the means and SE for the continuous variables are given. The mean birth order in the sample was found to be 3 births. The maternal age at first birth was 21 years, while the mean was having more than 3 children. The mean for overall DDS was found to be about 2, revealing that, on average, a child consumed 2 food types/groups, out of the 7.

**Table 2:** Descriptive statistics of the study sample (*n* = 1102): continuous variables (means and standard error)

Characteristics	Mean	SE
**Child (individual) level**		
Birth order	3.29	0.11
**Mother level**		
Age at first birth	21.11	0.15
Parity (number of children ever born)	3.38	0.11
Autonomy (Empowerment)^a^	3.34	0.1
**Household level**		
Number of children under 5 years	2.33	0.07
**Dietary diversity**		
Dietary diversity score (7 food groups)^b^	2.26	0.06
DDS by child age-group		
6–11 months	1.76	0.08
12–17 months	2.54	0.10
18–23 months	2.48	0.12

^a^The mother’s empowerment index was constructed based upon her participation in various decisions and her opinion or attitude towards domestic violence on the number of circumstances that justified beating

^b^Dietary diversity score categorises all food items, consumed by a child (6–23 months old) over the 24 h immediately preceding the survey, into 7 food groups, as recommended by the WHO. It included: (1) staples (grains/cereals, roots and tubers), (2) legumes and nuts, (3) dairy products (milk, yogurt and cheese), (4) flesh foods (meat, fish, poultry and liver/organ meats), (5) eggs, (6) vitamin A-rich fruits and vegetables; and (7) other fruits and vegetables

**Table 3:** Descriptive statistics of the study sample (*n* = 1102): categorical variables (frequency and percentages)

Characteristics	f	%
**Child (individual) level**		
Age		
6–11 months	400	33.6
12–17 months	407	41.0
18–23 months	295	25.4
Sex		
Male	559	50.2
Female	543	49.8
Size of child at birth		
Small or < average	224	20.7
Average or > average	870	79.3
Very large	2	0.0
Still breastfeeding		
Yes	840	75.1
No	262	24.9
**Mother level**		
Age		
15–24 years	349	33.7
25–34 years	590	53
35 years and above	163	13.4
Level of education		
No education	577	53.3
Primary	172	16.9
Secondary	220	20.6
Higher	133	9.2
Employment status		
Unemployed	908	82.7
Employed	194	17.3
Received services/information from LHW on MCH		
Yes	35	14.1
No	204	85.9
Number of ANC visits received		
At least 4 visits or more	461	43.0
Less than 4 visits	639	57.0
**Household level**		
Exposure to mass media		
Yes	911	81.2
No	187	18.8
Wealth index		
Poorest	223	20.6
Poorer	223	20.1
Middle	226	19.5
Richer	220	23.5
Richest	210	16.3
**Community level**		
Place of residence		
Urban	468	31.3
Rural	634	68.7
Region		
Punjab	306	57.8
Sindh	233	22.3
Balochistan	167	4.7
Khyber Paktunkhwa	227	14.1
Islamabad (ICT)	62	0.4
Gilgit Baltistan	107	0.8

Table 3 indicates the frequency and percentages of the categorical variables. Regarding child-level characteristics, 41% of children were 12–17 months of age, 33.6% were 6–11 months and 25.4% were 18–23 months. Half of the children were male. In most cases (75%), the children were still breastfed. Regarding maternal factors, 53% of mothers were in the age-group 25–34 years, approximately 34% were 15–24 years and 13.4% were 35 years and above. Most of the mothers were uneducated (53%) and unemployed (83%), made less than 4 antenatal visits (57%) and did not receive any information/services from an LHW on maternal and child health (86%). Of the household-level characteristics, 81% of households have exposure to mass media while 16.3% belong to the richest and approximately 21% to the poorest wealth quintile. Regarding community-level characteristics, 68.7% reside in rural areas and mainly in Punjab province (57.8%). A very few respondents live in Islamabad (0.4%) and Gilgit Baltistan (0.8%).

### Bivariate analysis

Simple linear regression analyses were performed to establish the associations between infants’ and young children’s dietary diversity and various sociodemographic variables, where R^2^, β (SE) and *p*-values were determined. A statistically significant association was seen for child age, breastfeeding status, maternal age and age at first birth, empowerment/autonomy, level of education, information/services received from LHWs on maternal and child health, number of ANC visits conducted, number of children under 5, wealth index, place of residence and region (Table 4).

**Table 4:** Bivariate simple linear regression of infants’ and children’s dietary diversity and its associated factors (*n* = 1102)

Characteristics	Dietary diversity		
	R^2^	β (SE)	*p*-value
**Child (individual) level**			
Age			
6–11 months			
12–17 months	0.05	0.47 (0.08)	<0.01
18–23 months	0.01	0.29 (0.10)	<0.01
Birth order	0.00	0.01 (0.02)	0.51
Sex			
Male	0.00	−0.001 (0.87)	0.99
Female			
Size of child at birth			
Small or < average			
Average or > average	0.00	0.12 (0.10)	0.23
Very large	0.00	2.01 (3.47)	0.56
Still breastfeeding			
Yes	0.04	−0.71 (0.09)	<0.01
No			
**Mother level**			
Age			
15–24 years			
25–34 years	0.01	0.31 (0.08)	<0.01
35 years and above	0.00	−0.005 (0.13)	0.97
Age at first birth	0.01	0.03 (0.01)	<0.01
Parity (number of children ever born)	0.00	−0.009 (0.02)	0.64
Autonomy (empowerment)	0.01	−0.04 (0.02)	<0.01
Level of education			
No education			
Primary	0.00	−0.07 (0.11)	0.52
Secondary	0.00	0.10 (0.11)	0.33
Higher	0.03	0.94 (0.15)	<0.01
Employment status			
Unemployed			
Employed	0.00	−0.21 (0.11)	0.07
Received services/information from LHW on MCH			
Yes	0.03	0.71 (0.24)	<0.01
No			
Number of ANC visits received			
At least 4 visits or more	0.00	−0.18 (0.09)	0.04
Less than 4 visits			
**Household level**			
Number of children under 5 years	0.01	−0.08 (0.04)	<0.01
Exposure to mass media			
Yes	0.00	−0.07 (0.11)	0.54
No			
Wealth index			
Poorest			
Poorer	0.01	−0.40 (0.11)	<0.01
Middle	0.00	−0.07 (0.11)	0.52
Richer	0.01	0.11 (0.10)	0.28
Richest	0.03	0.73 (0.12)	<0.01
**Community level**			
Place of residence			
Urban	0.01	0.37 (0.09)	<0.01
Rural			
Province/Region			
Punjab	0.00	0.14 (0.08)	0.09
Sindh	0.00	−0.001 (0.10)	0.99
Balochistan	0.01	−0.67 (0.20)	<0.01
Khyber Paktunkhwa	0.00	−0.07 (0.13)	0.58
Islamabad (ICT)	0.00	0.69 (0.74)	0.35
Gilgit Baltistan	0.00	0.07 (0.49)	0.88

### Multivariate analysis

Table 5 presents the results of the multiple linear regression analysis for the three models after testing for multicollinearity using the VIF. In Model A, children’s age was found to be significantly associated with dietary diversity. It revealed that the children’s dietary diversity increased to 0.73 (95% CI: 0.54–0.92) and 0.59 (95% CI: 0.38–0.81) amongst children aged 12–17 months and 18–23 months respectively. However, a negative association was seen with children who were still breastfeeding. In Model B, mothers having higher education and receiving information/services from LHWs on maternal and child health showed a positive and significant association with dietary diversity, whereby DDS increased to 0.64 (95% CI: 0.17–1.10) for mothers who received information/services from LHWs. In Model C, where all factors were included, children in the middle age-group, children who were still breastfeeding, mothers’ education up to primary level and mothers who received information/services from LHWs on maternal and child health were found to be statistically significant predictors of infants’ and young children’s dietary diversity. Furthermore, the multivariate analysis (Model C) revealed that the children’s DDS increases to 0.56 (95% CI: 0.18–0.94) in children in the middle age-group (12–17 months). Nevertheless, amongst them, the DDS had a negative association with the children’s status of still breastfeeding and mothers’ primary level of schooling, whereas it had a positive association with children being in the middle age-group and mothers who received information/services from LHWs on maternal and child health. The reason for nonlinearity could be that the DDS is also not independent of other factors.

**Table 5:** Multivariate linear regression of infants’ and young children’s dietary diversity and its associated factors (*n* = 1102)

Characteristics	Model A^a^			Model B^a^			Model C^a^		
	β (SE)	CI (95%)	*p*-value	β (SE)	CI (95%)	*p*-value	β (SE)	CI (95%)	*p*-value
**Child (individual) level**									
Age									
6–11 months (Ref.)									
12–17 months	0.73 (0.09)	0.54–0.92	<0.01	0.57 (0.19)	0.19–0.94	<0.01	0.56 (0.19)	0.18–0.94	<0.01
18–23 months	0.59 (0.11)	0.38–0.81	<0.01	0.34 (0.22)	−0.09-0.78	0.12	0.28 (0.22)	−0.16-0.72	0.21
Still breastfeeding									
No (Ref.)									
Yes	−0.63 (0.09)	−0.82-(−0.43)	<0.01	−0.86 (0.19)	−1.24-(−0.48)	<0.01	−0.89 (0.199)	−1.28-(−0.50)	<0.01
**Mother level**									
Age									
15–24 years (Ref.)									
25–34 years				−0.11 (0.18)	−0.47-0.25	0.54	−0.08 (0.18)	−0.44-0.29	0.67
35 years and above				−0.29 (0.27)	−0.82-0.24	0.28	−0.29 (0.27)	−0.82-0.24	0.28
Age at first birth				0.02 (0.02)	−0.03-0.07	0.38	0.01 (0.02)	−0.03-0.06	0.49
Level of education									
No education (Ref.)									
Primary				−0.53 (0.25)	−1.02-(−0.04)	0.04	−0.68 (0.26)	−1.02-(−0.16)	<0.01
Secondary				0.12 (0.19)	−0.27-0.51	0.54	−0.01 (0.22)	−0.45-0.43	0.97
Higher				0.61 (0.29)	0.03–1.20	0.04	0.44 (0.33)	−0.21-1.08	0.18
Received services/information from LHW on MCH									
No (Ref.)									
Yes				0.64 (0.24)	0.17–1.10	<0.01	0.57 (0.24)	0.09–1.04	0.02
Number of ANC visits received									
Less than 4 visits (Ref.)									
At least 4 visits or more				−0.01 (0.16)	−0.33-0.30	0.94	−0.02 (0.16)	−0.34-0.29	0.88
**Household & Community level**									
Number of children under 5 years							0.01 (0.07)	−0.12-0.15	0.83
Place of residence									
Rural (Ref.)									
Urban							−0.09 (0.23)	−0.54-0.34	0.68
Model for good fit (R^2^)	0.10			0.17			0.20		

^a^Model A included the dietary diversity and child-level characteristics. In Model B, the maternal level characteristics were also added with child-level factors, and in Model C, household and community-level characteristics were also included in addition to child and maternal-level factors. Moreover, multiple linear regression was conducted using those variables that were found to be significantly associated with infants’ and young children’s dietary diversity in the bivariate analysis

## Discussion

An emphasis on dietary diversity, composed of a number of food items and food groups, amongst children is essential to ensure adequate and balanced nutrients for a child’s growth and development [33, 34]. Dietary diversity is generally assessed using a simple tool, the Dietary Diversity Score (DDS), equivalent to the number of specified food groups consumed during the previous 24 h [25]. In particular, dietary diversity does not merely capture the variety of foods consumed, but also indicates their relative distribution and parental adherence to the recommended dietary patterns. This research has investigated the association between the dietary diversity of infants and young children (aged 6–23 months) and the various factors associated with dietary practices in Pakistan. The overall results showed a positive relationship between children’s dietary diversity and sociodemographic variables, after accounting for the influences of individual, maternal, household and community-level factors. This implies that better sociodemographic conditions promote the consumption of a variety of foods among infants and young children. These findings are similar to those of previously conducted research [35, 36].

On average, infants’ and young children’s diets in Pakistan consisted of two food groups. Staples (grains/cereals, roots and tubers) and dairy products (milk, yogurt and cheese) were found to be the most common foods groups consumed. The reported mean for the DDS was found to be consistent with that for other countries, such as Ethiopia [16]. A variation was also observed regarding consumption of the 7 food groups across age-groups of children. Findings revealed that the variety of consumption of food groups increased in the middle age-group (12–17 months), and then reduced in the older age-group (18–23 months). This finding is similar to the study conducted in Ethiopia, which revealed that mothers had relatively good dietary diversity practices for younger children, which decreased in the older age-group [37]. An explanation for this could be that children in the older age-groups are consuming other foods, high in fats and sugar. Nevertheless, the multivariate analysis showed that an increase in the age of children is significantly associated with dietary diversity, which is similar to the situation documented in previous studies [36, 38, 39]. Moreover, a study conducted with children aged 6–12 months in the UK revealed that patterns of children’s dietary diversity are linked to maternal and household-level characteristics [40].

Comparable findings were obtained in Mali, where researchers observed a significant relationship between children’s dietary diversity and socioeconomic status [35]. Their results showed that children’s dietary diversity increased with higher socioeconomic status. Moreover, a difference was seen between the dietary diversity of children residing in urban and rural areas, with urban households having a consistently higher diversity in diet than rural areas. Likewise, a significant relationship was observed between dietary diversity and the services/information received from LHWs on maternal and child health. This finding is consistent with a study showing that utilisation of health services helps mothers to improve their children’s feeding practices [41].

Other similar studies also suggest a positive relationship between dietary diversity and socioeconomic factors. For example, a study from the southern Andes highlighted that dietary diversity is greater in households with higher socioeconomic status [20]. A difference was also observed between poorer and wealthier households in urban areas, whereby the poorer households consumed less diverse diets, due to their poor intake of dairy products, meat and vegetables [40]. A similar study conducted with preschool children in Ghana and Malawi also documented a difference in dietary diversity amongst households of varied socioeconomic status [42]. Summarising the above, the positive association between dietary diversity and various aspects of sociodemographic characteristics demonstrates that infants’ and young children’s dietary diversity in Pakistan is highly influenced by contextual factors. However, along with sociodemographic variables, there is a need to explore the association of dietary diversity with structural aspects as well, such as food security, including the availability, accessibility and affordability of nutritious foods at the household level. This is also suggested by the multi-country data analysis (India, the Philippines, Bangladesh, Egypt, Mali, Ghana, Malawi, Kenya, Mexico and Mozambique), where the association between household dietary diversity, socioeconomic status and food security was explored [43].

This study has its own strengths and limitations. An important strength is the use of the 7 food groups to measure a dietary diversity score as recommended by the WHO. Moreover, nationally representative data from the PDHS was used to investigate the dietary diversity practices of infants and young children aged 6–23 months in Pakistan. Nevertheless, this study has some limitations. One is restricting the analysis to mothers having a last-born child aged 6–23 months only, which yielded a sample size of 1102 children. Another limitation worth mentioning is the 24-h recall method that was used in dietary data collection in the DHS. The weakness of this method lies in the fact that, due to cognitive challenges, mothers may not be able to recall or report their child’s food consumption precisely. Cognitive challenges may include lack of knowledge and memory (forgetfulness) and the situation of the interview [44]. Furthermore, the 24-h recall method has a tendency to underestimate the food intake by about 10% as compared to the observed intake [45]. Nevertheless, the DHS had an experienced interviewer probing to overcome these cognitive challenges. The lack of seasonality information could also be a limitation; the seasonal variation was almost negligible during the data-collection phase. Additionally, the lack of information on some important variables, such as seasonality of consumption, cooking methods used, and detailed information about portion size, was also a limitation.

## Conclusion

Dietary diversity has a modest, nevertheless statistically significant, relationship with sociodemographic characteristics in Pakistan. The findings indicate a need to target mothers, particularly those of lower sociodemographic status, through formal health-education programmes, advocacy and awareness-raising campaigns to foster their children’s growth with a more diverse diet. There is a need for practical efforts to change the behaviour of communities through community mobilisation activities. The services of LHWs will be beneficial for continuous health education to encourage the consumption of more diverse foods amongst infants and young children. Moreover, a high level of political commitment is also required to prioritise the need to end food insecurity, poverty and malnutrition at policy level to promote the healthy growth of children.

## Abbreviations

DDS: Dietary Diversity Score
LHW: Lady Health Worker
NNS: National Nutrition Survey
PDHS: Pakistan Demographic and Health Survey
SE: Standard Error
VIF: Variance Inflation Factor
WHO: World Health Organisation
